# PD1 is highly expressed in diffuse large B-cell lymphoma with hepatitis B virus infection

**DOI:** 10.1371/journal.pone.0180390

**Published:** 2017-06-29

**Authors:** Zhihe Liu, Siyun Li, Yingmin Liu, Wei Guo, Ou Bai

**Affiliations:** 1 Department of Hematology, the First Hospital of Jilin University, Changchun, China; 2 Department of Pediatrics, the Affiliated Women & Children Hospital of Qingdao University Medical School, Qingdao, China; Centre de Recherche en Cancerologie de Lyon, FRANCE

## Abstract

**Objective:**

The purpose of this study was to determine the association between PD1 expression and the clinical prognosis of diffuse large B-cell lymphoma (DLBCL) co-occurring with hepatitis B virus (HBV) infection.

**Methods:**

A total of 165 patients presented with newly diagnosed and untreated DLBCL at the First Hospital of Jilin University, Changchun, China, between 2011.01 and 2014.12. Complete clinical information was available for 152 of these 165 patients. We retrospectively reviewed the results of HBV serum marker assays and the clinical information of these 152 DLBCL patients from our hospital database; eventually, only 51 patients were enrolled in this study, and these 51 patients received the PD1 test item.

**Results:**

**①** The incidence of HBsAg prevalence was 13.2% (20/152) in this study; **②** The incidence of PD1 expression in the HBsAg^+^ group was 4.3-fold higher than that in the HBsAg^**—**^group (40.0% *vs* 9.4%; *P* = 0.010); **③** The clinical information, including sex, age, clinical stage, IPI, molecular subtype and chemotherapy status, was analyzed between the HBsAg^+^ and HBsAg^**—**^groups, but there were no significant differences between the two groups; **④** The median OS and PFS of the patients in the HBsAg^+^ group were 36.5 months and 12 months, respectively; however, the median OS and PFS of patients in the HBsAg^**—**^group were not reached (*P* = 0.033) and 32 months (*P* = 0.049), respectively; and **⑤** The median OS and PFS of PD1-positive patients in the HBsAg^+^ group were the worst (24 months and 9 months, respectively), whereas the median OS and PFS of PD1-negative patients in the HBsAg^**—**^group were the best (not reached and 32 months, respectively).

**Conclusions:**

Compared with patients in the HBsAg^**—**^group, the incidence of PD1 expression was significantly higher in the HBsAg^+^ group, and the median OS and PFS times were the worst in PD1-positive patients in the HBsAg^+^ group. These results indicated that the dismal prognosis of patients with HBsAg^+^ may be related to the high rate of PD1 expression. Thus, a targeted PD1 treatment strategy may improve the prognosis of HBsAg^+^ DLBCL patients.

## Introduction

Hepatitis B virus (HBV) infection remains an important public health problem, as nearly 2 billion people are currently infected with HBV. Worldwide, 360 million people have chronic HBV infections; this number includes 93 million people in China [1–3]. Based on the data resulting from a global analysis of hepatitis B surface antigen (HBsAg) prevalence by country conducted in 2000, the prevalence of HBsAg in China was 5.71%, which was higher than that in other countries in the Western Pacific, such as Australia (0.42%), Japan (0.57%), Malaysia (0.77%), New Zealand (4.05%), the Republic of Korea (3.96%) and Singapore (3.31%) [4]. Therefore, China currently remains a region in which HBV is endemic. The financial expenditure imposed by the HBV burden is high, and approximately 300,000 deaths are caused each year by HBV-related diseases [5].

It has already been proven that HBV infection can increase the risks of non-Hodgkin’s lymphoma, especially DLBCL [6–8]. In addition, some studies have suggested that the clinical prognosis of patients with HBV-associated DLBCL has been poor in the era of rituximab [9]. To improve the clinical prognosis of patients with HBV-associated DLBCL, more effective treatment strategies are urgently needed.

Currently, immunotherapy has made remarkable progress in the treatment of tumors. The programmed cell death 1/programmed cell death ligand 1 (PD1/PD-L1) cell signaling pathway is closely related to the functioning of the immune system, which is currently a research hotspot in the field of tumor immunotherapy. The activation of the PD1/PD-L1 cell signaling pathway can suppress the function of the immune system and contribute to the immune escape of cancer cells. Some studies have indicated that tumor progression and inferior prognoses are associated with the activation of the PD1/PD-L1 pathway, which notably inhibits the immune system [10–12]. It was also identified in preclinical trials that the therapeutic effect of EBV+DLBCL can be improved by the blockade of the PD1/PD-1 pathway with an anti-PD1 monoclonal antibody [13,14]. Given that both EBV+DLBCL and HBV+DLBCL are associated with viral infections, we suspect that the PD1/PD-L1 cell signaling pathway may play an important role in the clinical prognosis of DLBCL patients with concurrent HBV infection.

Based on the assumption above, we first retrospectively reviewed the results of HBV serum marker assays and the clinical information of 152 out of 165 patients with newly diagnosed and untreated DLBCL from our hospital database. According to the results of the HBV serum marker assays, only 51 patients were included in this study. Subsequently, these 51 patients received PD1 test items, and ultimately, we systematically investigated the influence of PD1 expression on the clinical prognosis of DLCBL patients with concurrent HBV infection.

## 1 Materials and methods

### 1.1 Patients and samples

A total of 165 cases of newly diagnosed and untreated DLBCL-NOS were reviewed at the First Hospital of Jilin University from 2011.01 to 2014.12. Complete clinical information was available for 152 of these 165 DLBCL-NOS patients. In this study, EBV+DLBCL patients and hepatitis C virus-positive DLBCL (HCV+DLBCL) patients were excluded, and all patients received first-line therapies, such as CHOP, R-CHOP and CHOP/R-CHOP-like regimens, in this study. All patients enrolled were reclassified by hematologists (YM.L. and O.B.) based on the World Health Organization classification [15] and Hans’ algorithm [16], and the clinical stages of all patients were in line with the Ann Arbor staging system [17]. The clinical information from all patients was collected from our hospital database. This study was performed in accordance with the Declaration of Helsinki, and this study was approved by the ethics review committee of the First Hospital of Jilin University. Written informed consent was obtained from each patient or their legal guardians. The authors did not have access to information that could identify individual participants during or after the data collection, as the information that could identify the patients was processed in our hospital database.

### 1.2 HBV serum marker assays

All patients with lymphoma were required to undergo HBV serum marker assays utilizing ELISA kits provided by SHANGHAI KEHUA BIO-ENGINEERING Co., Ltd. in the Department of Laboratory Medicine, First Hospital, Jilin University, before treatments. This test assessed the expression levels of hepatitis B surface antigen (HBsAg), hepatitis B surface antibody (HBsAb), hepatitis B e antigen (HBeAg), hepatitis B e antibody (HBeAb) and hepatitis B core antibody (HBcAb). We retrospectively reviewed the results of the HBV serum marker assays of those 152 DLBCL patients with complete clinical information from our hospital database; overall, 20 patients were at least HBsAg^+^ and 31 cases were only HBsAb-positive or fully negative for all HBV serum markers Table 1. At present, patients with HBsAg^+^ are generally defined as being infected with HBV; therefore, the 20 HBsAg^+^ patients were assigned to the HBsAg^+^ group, and the 31 cases with only HBsAb-positive results or negative results for all HBV serum markers were assigned to the HBsAg^**—**^group. For those HBsAg-positive patients, the DNA copy number was detected by real-time quantitative polymerase chain reaction (RT-PCR) before each cycle of chemotherapy. Those patients received antiviral therapy (Entecavir) until the DNA copy number was less than 1000 IU/ml if the DNA copy number was more than 1000 IU/ml before chemotherapy. Antiviral therapy was administered for at least six months after the end of chemotherapy for DLBCL patients in the HBsAg^+^ group.

**Table 1:** Results of the HBV serum marker assays for 152 patients.

Group	HBV serum markers	n (%)
HBsAg^+^	HBsAg is at least positive	20 (13.2%)
HBsAg^—^	HBsAg^—^, HBsAb^+^, HBeAg^—^, HBeAb^—^, HBcAb^—^	27 (17.8%)
	HBsAg^—^, HBsAb^—^, HBeAg^—^, HBeAb^—^, HBcAb^—^	4 (2.6%)
Others	HBsAg^—^, HBsAb^+^, HBeAg^—^, HBeAb^+^, HBcAb^+^	26 (17.1%)
	HBsAg^—^, HBsAb^+^, HBeAg^—^, HBeAb^—^, HBcAb^+^	32 (21.1%)
	HBsAg^—^, HBsAb^+^, HBeAg^—^, HBeAb^+^, HBcAb^—^	5 (3.3%)
	HBsAg^—^, HBsAb^—^, HBeAg^—^, HBeAb^+^, HBcAb^+^	8 (5.3%)
	HBsAg^—^, HBsAb^—^, HBeAg^—^, HBeAb^—^, HBcAb^+^	30 (19.7%)

n: number.

### 1.3 CD3 and PD1 double staining

**Fig 1:**
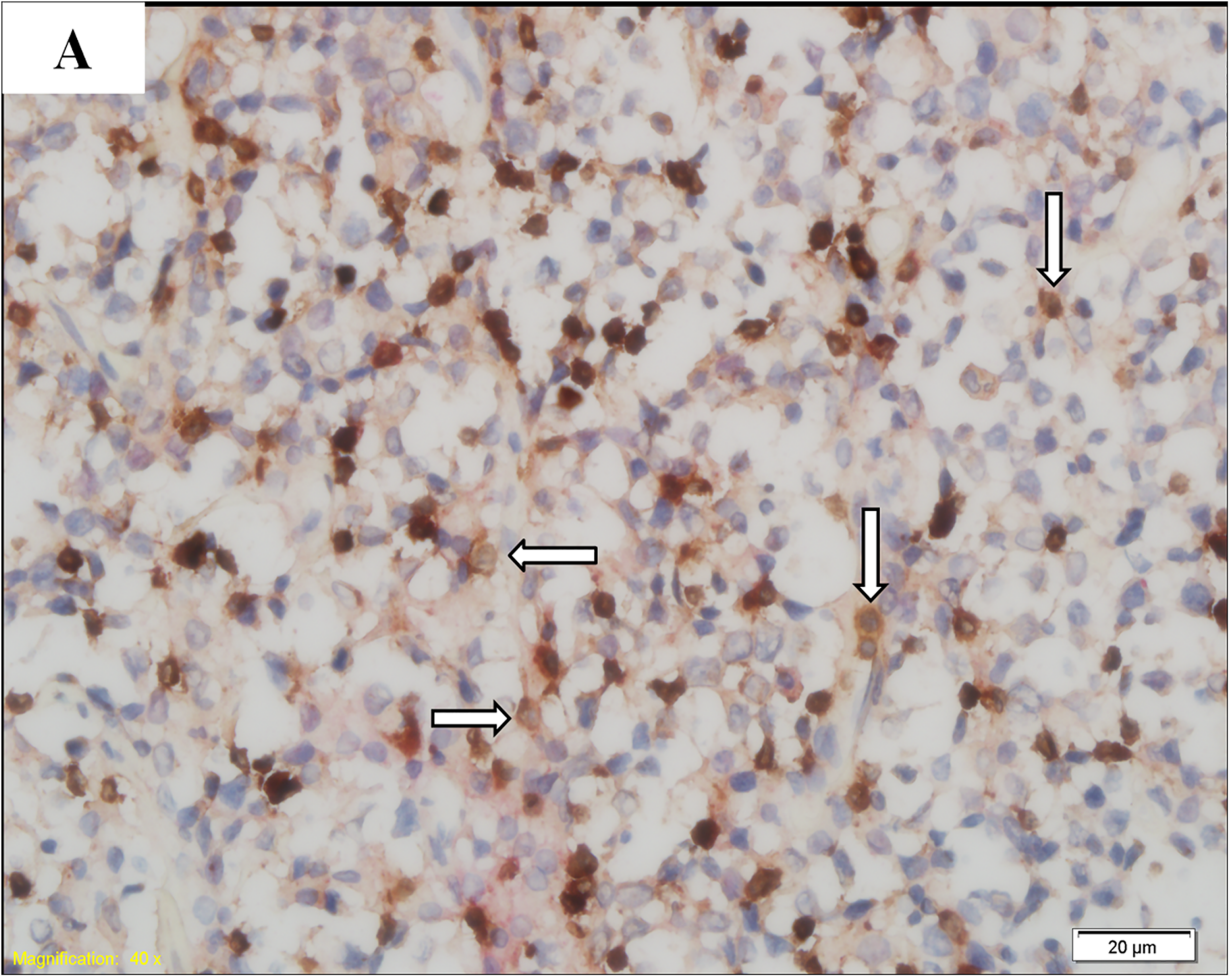
PD1-negative DLBCL. T lymphocytes (arrow) are positive for CD3 (brown) and negative for PD1 (red).

**Fig 2:**
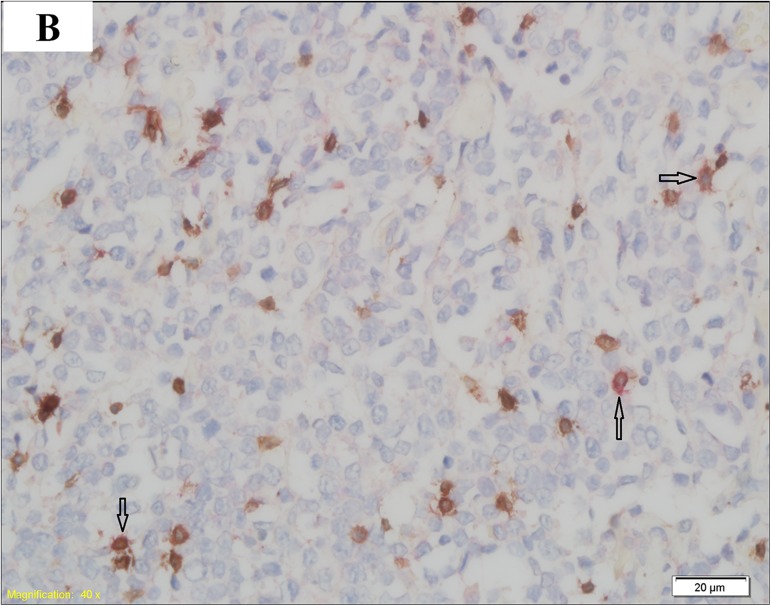
PD1-positive DLBCL. T lymphocytes (arrow) are positive for both CD3 (brown) and PD1 (red).

In this study, we obtained 2 slices of 5-μm thick formalin-fixed and paraffin-embedded (FFPE) lymphoma tissue sections from each patient in our hospital pathology department. Then, CD3 and PD1 double staining was performed in the sections obtained from 20 DLBCL patients in the HBsAg^**+**^ group and 31 DLBCL patients in the HBsAg^**—**^group. Each FFPE tissue section was first stained with a CD3 primary antibody (purchased by Tianjin Yongxinkang BIO-ENGINEERING Co Ltd, LZM-0109) to identify T lymphocytes, followed by staining with a PD1 primary antibody (purchased by Tianjin Yongxinkang BIO-ENGINEERING Co Ltd, LZM-0113) to determine which T lymphocytes expressed PD1. One hundred cells were counted in each FFPE tissue section. PD1 expression was defined as positive if more than 10 CD3-positive cells were stained with PD1. The results of PD1 expression are shown in (Figs 1 and 2).

### 1.4 Follow-up

All 51 DLBCL patients were effectively followed up to September 2016, and the median follow-up time of all 51 patients was 30.0 months. OS was defined from diagnosis to death or the last follow-up, and PFS was defined from diagnosis to disease progression, death, or last follow-up.

### 1.5 Statistics

SPSS 20.0 software was used for the statistical analysis. OS and PFS were analyzed using the Kaplan-Meier method. Clinical information, protein expression of PD1 in the HBsAg^+^ and HBsAg^**—**^groups were compared using Χ^2^ tests. *P*≤0.05 was considered statistically significant.

## 2 Results

### 2.1 Clinical information

To compare the clinical prognosis of patients in the HBsAg^+^ and HBsAg^—^groups, we retrospectively analyzed the clinical information of the 51 patients enrolled in this study (S1 File). The median age of all patients was 53 years (22–89 years), and there were no significant differences between the two groups in terms of sex (*P* = 0.927), age (*P* = 0.078), clinical stage (*P* = 0.146), IPI (*P* = 0.865), molecular subtype (*P* = 0.572) and chemotherapy regimens (*P* = 0.244) Table 2. However, compared to patients in the HBsAg^—^group, those in the HBsAg^+^ group were older at the time of disease onset (>60 years; 50.0% *vs* 25.8%). The patients in the HBsAg^+^ group also displayed more advanced clinical stages (Ⅲ/Ⅳ; 75.0% *vs* 54.8%), and the rate of patients receiving rituximab-combined chemotherapy in the HBsAg^+^ group was lower (35.0% *vs* 51.6%).

**Table 2:** Clinical information of patients in this cohort.

	HBV^+^ group (n = 20)	HBV^—^group (n = 31)	*P* value
**Sex**			
male	12 (60.0%)	19 (61.3%)	0.927
female	8 (40.0%)	12 (38.7%)	
**Age**			
≥60	10 (50.0%)	8 (25.8%)	0.078
<60	10 (50.0%)	23 (74.2%)	
**Stage**			
Ⅰ/Ⅱ	5 (25.0%)	14 (45.2%)	0.146
Ⅲ/Ⅳ	15 (75.0%)	17 (54.8%)	
**IPI**			
>2	6 (30.0%)	10 (32.3%)	0.865
≤2	14 (70.0%)	21 (67.7%)	
**Molecular subtype**			
GCB	8 (40.0%)	10 (32.3%)	0.572
non-GCB	12 (60.0%)	21 (67.7%)	
**Chemotherapy**			
Rituximab-combined	7 (35.0%)	16 (51.6%)	0.244
Rituximab-free	13 (65.0%)	15 (48.4%)	

### 2.2 OS and PFS of patients in the HBsAg^+^ and HBsAg^—^groups

**Fig 3:**
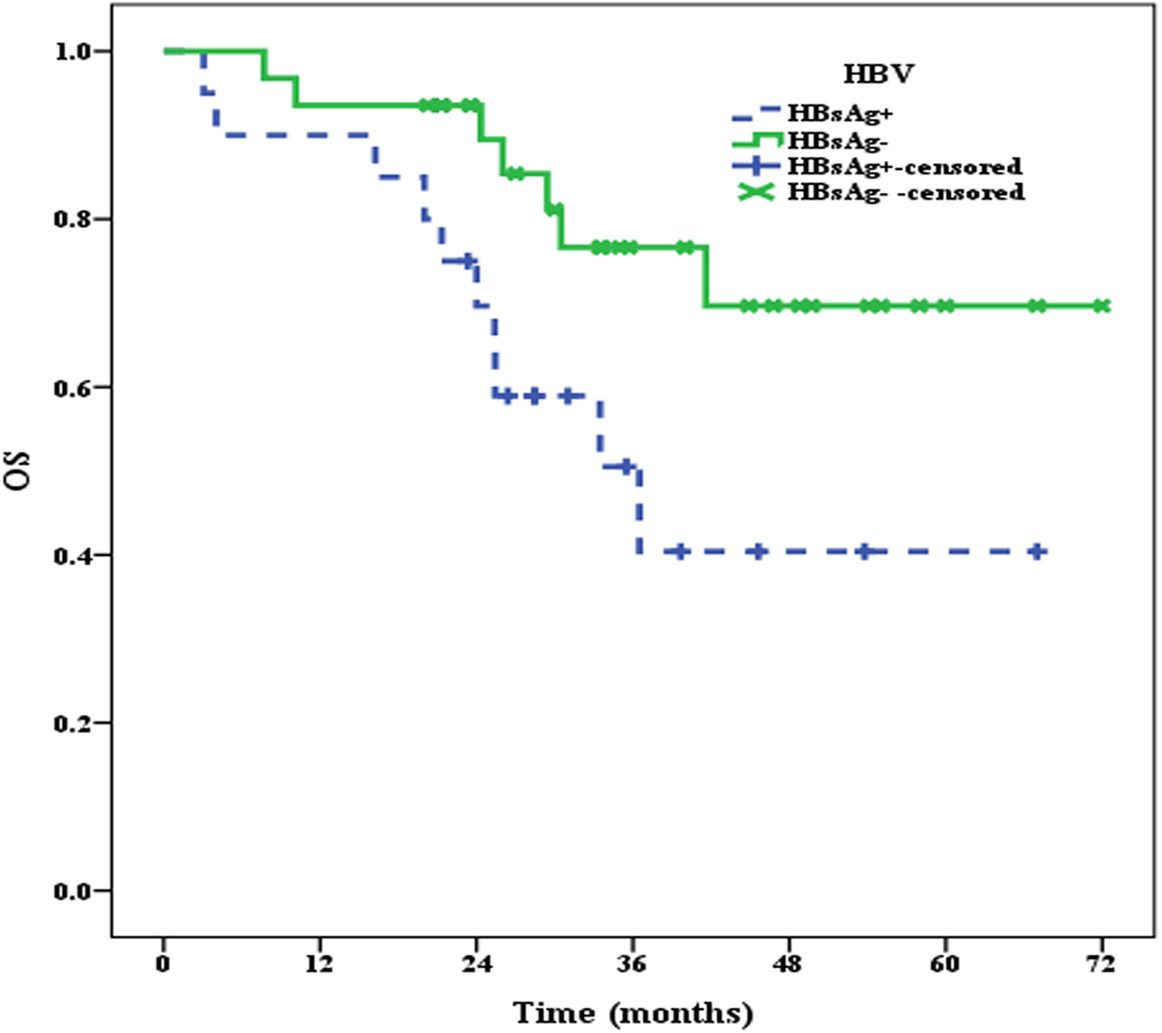
The median OS of patients in the HBsAg^+^ and HBsAg^—^groups.

**Fig 4:**
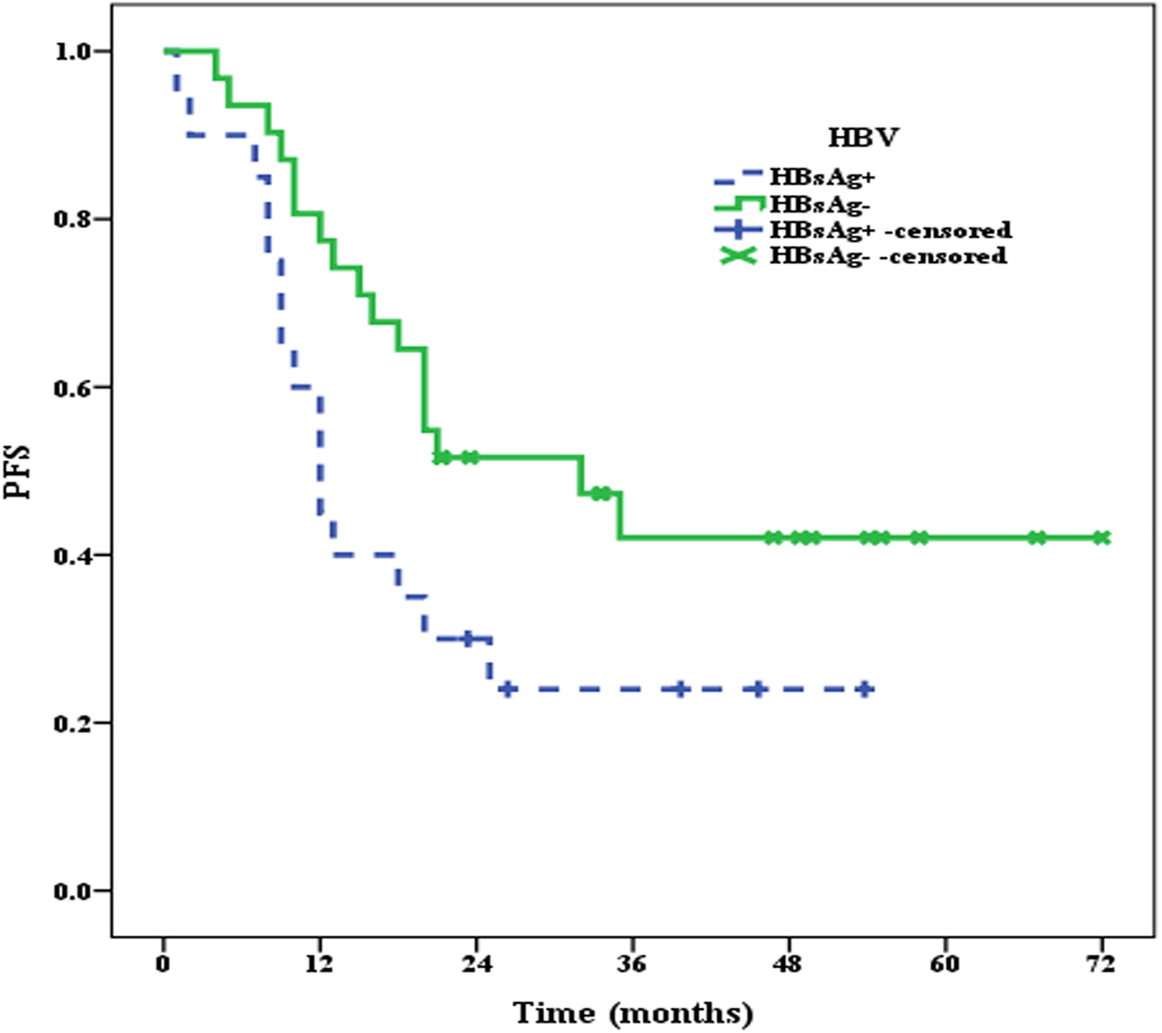
The median PFS of patients in the HBsAg^+^ and HBsAg^—^groups.

All 51 patients were effectively followed up until September 2016, and the median follow-up time was 30.0 months. At the end of follow-up, 10 patients in the HBsAg^+^ group and 7 patients in the HBsAg^**—**^group had died. The median OS of patients in the HBsAg^+^ group was 36.5 months, whereas the median OS of patients in the HBsAg^**—**^group was not reached, and there were significant differences between the two groups (*P* = 0.033) (**Fig 3**). Moreover, the median PFS of patients in the HBsAg^+^ group was only 12 months, which was significantly shorter (*P* = 0.049) than that in the HBsAg^**—**^group (32 months) (**Fig 4**).

### 2.3 PD1 expression

**Fig 5:**
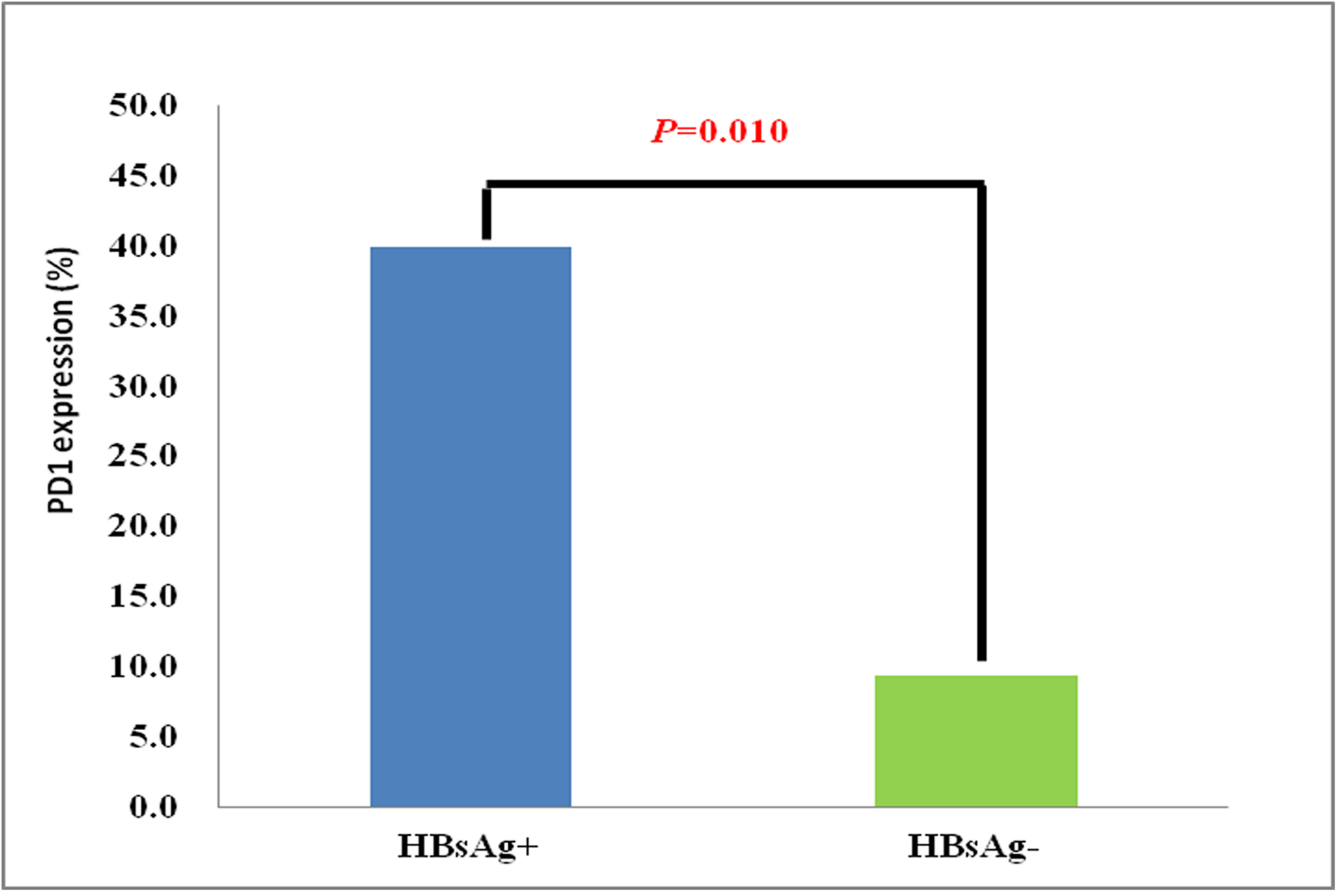
PD1 expression in the HBsAg^+^ and HBsAg^—^groups.

The clinical prognosis of patients in the HBsAg^+^ group was worse than that in the HBsAg^**—**^group. Moreover, there were no significant differences in the clinical information of patients in the two groups. Therefore, we speculated that PD1 expression may be closely associated with the adverse prognosis of patients in the HBsAg^+^ group. Thus, all patients underwent the PD1 test item in this study. On the basis of the cutoff of PD1 expression, 40.0% (8/20) patients were considered to be PD1 positive in the HBsAg^+^ group, and less than 10 percent of patients (9.4%; 3/31) were identified as PD1 positive in the HBsAg^**—**^group, with significant differences between the two groups (*P* = 0.010) (**Fig 5**).

### 2.4 Associations of PD1 with OS and PFS of patients in the HBsAg^+^ and HBsAg^—^groups

**Fig 6:**
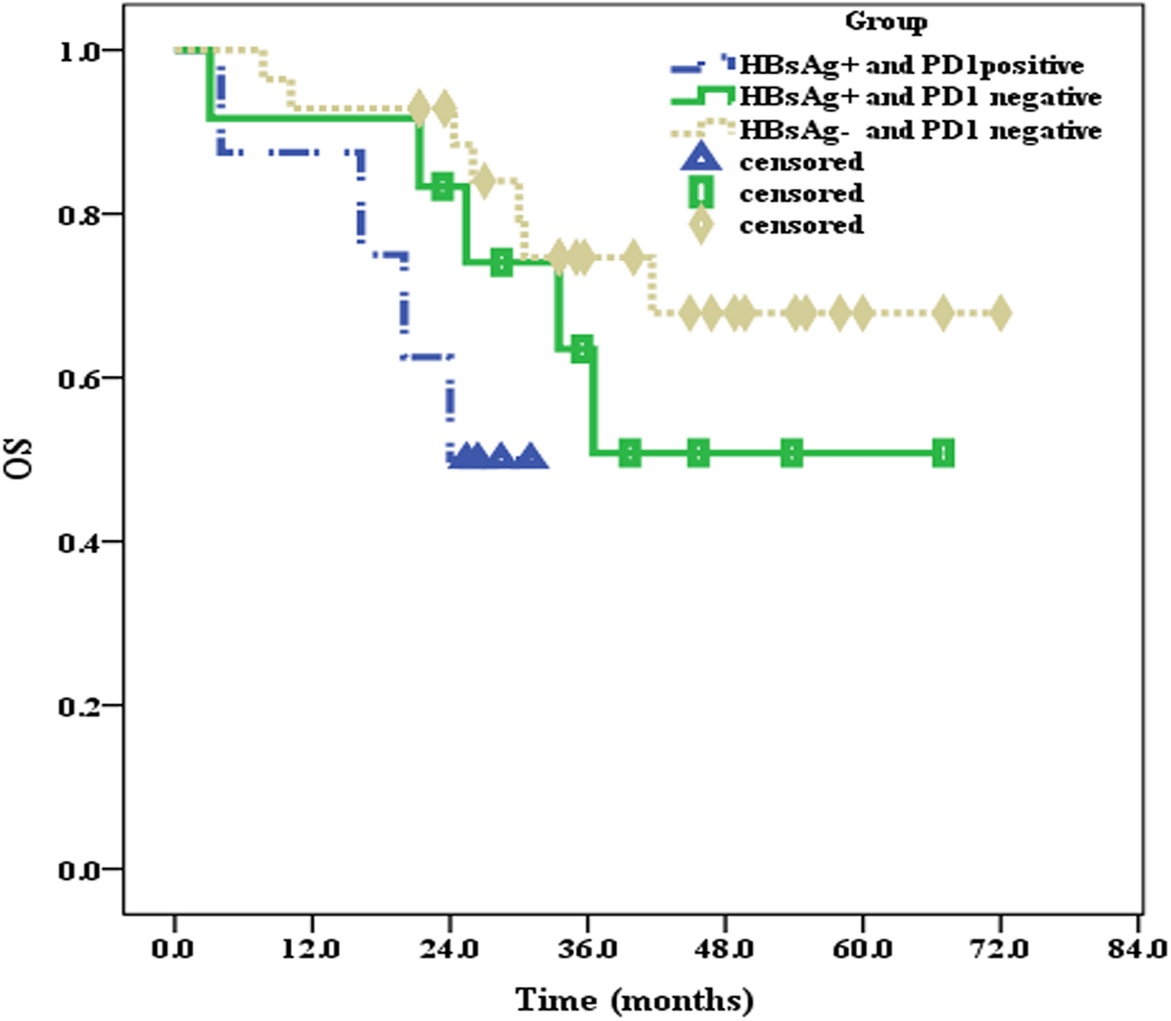
PD1 expression and the median OS of patients in the HBsAg^+^ and HBsAg^—^groups.

**Fig 7:**
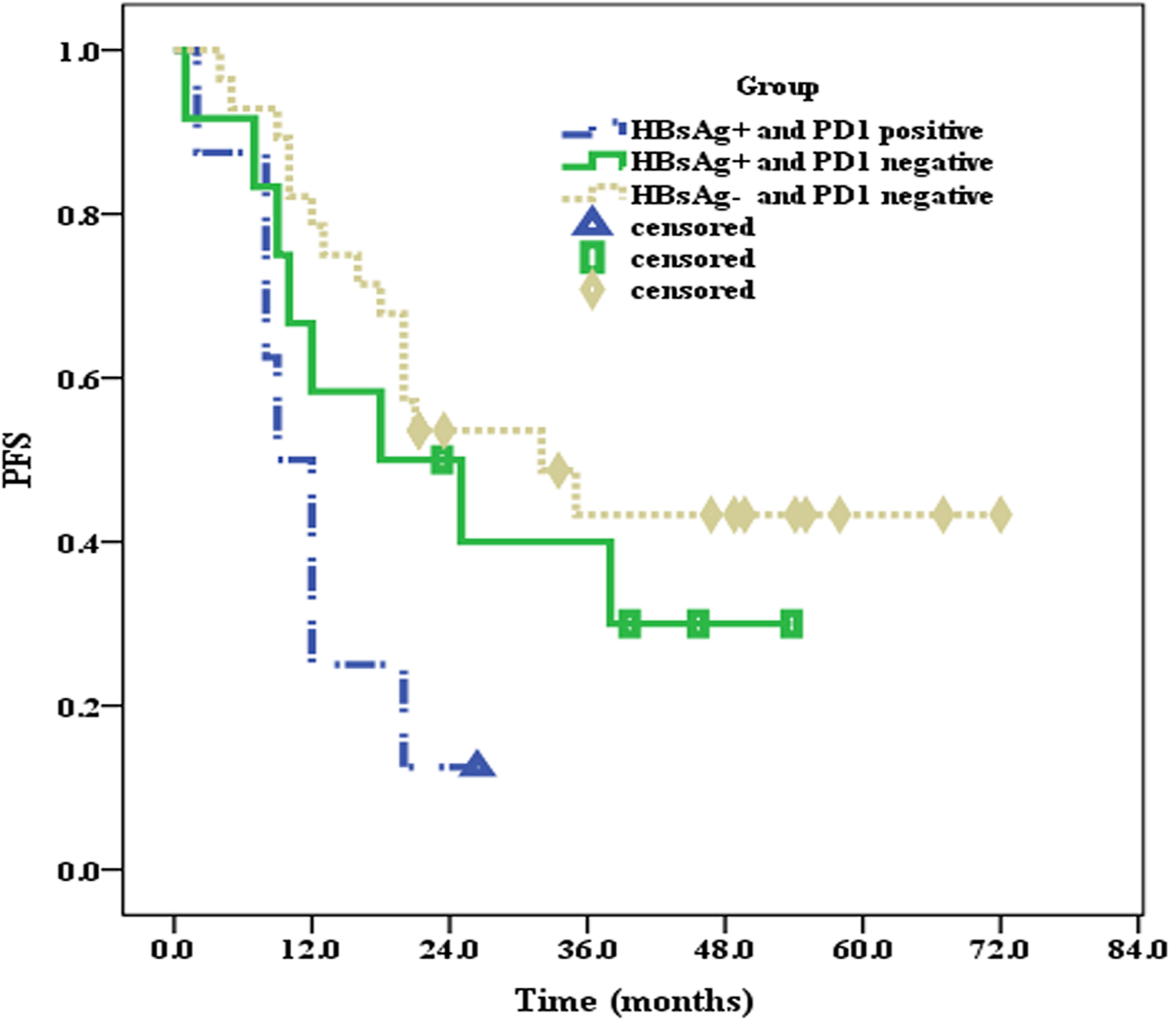
PD1 expression and the median PFS of patients in the HBsAg^+^ and HBsAg^—^groups.

Compared to patients in the HBsAg^**—**^group, the incidence of PD1 expression was higher than that in the HBsAg^+^ group. Thus, we further investigated the influence of PD1 expression on the clinical prognosis of DLBCL patients in the HBsAg^+^ and HBsAg^**—**^groups. Because there were only three PD1-positive patients in the HBsAg^**—**^group, the clinical prognoses of these three patients were not statistically analyzed. Until the end of the follow-up, the median OS of PD1-positive patients in the HBsAg^+^ group was 24 months; however, the median OS values of PD1-negative patients in both the HBsAg^**—**^(*P* = 0.038) and HBsAg^+^ groups (*P* = 0.250) were not reached (**Fig 6**). The median PFS of PD1-positive patients in the HBsAg^+^ group was 9 months, the median PFS of PD1-negative patients in the HBsAg^+^ group was 18 months (*P* = 0.163), and the median PFS of PD1-negative patients in the HBsAg^**—**^group was 32 months (*P* = 0.005) (**Fig 7**).

## Discussion

In this study, we retrospectively reviewed the results of HBV serum marker assays and clinical information, such as age, sex, clinical stage, IPI, molecular subtype and chemotherapy status, of 152 DLBCL patients with complete clinical information available in our hospital database. We then examined the PD1 expression levels in 20 DLBCL patients from the HBsAg+ group and 31 DLBCL patients from the HBsAg—group and investigated the association between PD1 expression and the clinical prognosis of patients in this cohort. The results were as follows: ① The incidence of HBsAg was 13.2% in this study; ② PD1 was highly expressed in the HBsAg+ group, with an incidence of 40.0%; ③ The median OS and PFS of patients in the HBsAg+ group were shorter than those in the HBsAg—group; and ④ The median OS and PFS of PD1-positive patients in the HBsAg+ group was the shortest, whereas the median OS and PFS of PD-negative patients in the HBsAg—group was the best.

There were no significant differences in terms of clinical information between the two groups of patients in this study. However, when considering age (≥60 years), clinical stage (Ⅲ/Ⅳ stage) and chemotherapy regimens (rituximab-free), the number of patients presenting with these factors was higher in the HBsAg+ group than in the HBsAg—group. These results were consistent with the outcomes reported by us in a previous study [18]. Although these three factors may have a certain relationship with the poor prognosis of patients in the HBsAg+ group, they are not the most vital factors. Therefore, in this study, we further explored the PD1 expression levels and the relationship between PD1 expression and poor patient prognoses in the HBsAg+ group.

The PD1 expression levels in DLBCL patients in the HBsAg+ group was higher than the published data; however, the PD1 expression levels in DLBCL patients in the HBsAg—group was consistent with the previously published data [19]. This phenomenon can be well explained by the idea that chronic infection can increase PD1 expression levels in T cells. For example, T cells that are co-cultured with EBV+ lymphoma cell lines, including EBV+DLBCL lymphoma cells, show remarkably augmented PD1 expression [20,21].

In this study, the PD1 expression levels on TILs were examined using CD3 and PD1 double staining. The results indicated that PD1 expression, which was remarkably higher in the HBsAg+ group, may be involved in the adverse prognosis of patients in this group. The prognostic role of PD1 expression on tumor-infiltrating lymphocytes (TILs) was previously evaluated in patients with follicular lymphoma (FL), and the results suggested that PD1 expression on TILs was beneficial to the OS and PFS of patients with FL [19,22]. However, the relationship between PD1 expression on TILs and the prognosis of DLBCL, and especially the association between PD1 expression on TILs and the prognosis of DLBCL with HBsAg+, remains unclear. Accordingly, we investigated the relationship between PD1 expression on TILs and the prognosis of patients with HBsAg+ in this study. First, we analyzed the OS and PFS of patients in the HBsAg+ and HBsAg—groups, and the results showed that the prognosis of patients in the HBsAg+ group was worse than those in the HBsAg—group. We then further explored the effects of PD1 expression on the prognosis of patients in the HBsAg+ and HBsAg—groups. The results indicated that the median OS and PFS of PD1-positive patients in the HBsAg+ group was the worst. These results suggested that the poor prognosis of DLBCL patients with HBsAg+ may be strongly associated with PD1 expression on TILs.

The prognosis of DLBCL patients with HBsAg+ was inferior to that of patients without HBV infection even in patients receiving rituximab-based chemotherapy. Therefore, an urgent problem remains in how the survival of patients with HBsAg+ can be improved. In this study, we explored the levels of PD1 expression in patients with HBsAg+, and the results showed that PD1 was highly expressed in patients with HBsAg+. Some preclinical trials have reported that the therapeutic effects of treatment in patients with EBV+DLBCL can be improved by blocking the PD1/PD-1 cell signaling pathway with anti-PD1 monoclonal antibody [13,14]. Consequently, we believed that chemotherapy containing rituximab combined with anti-PD1 targeted therapies, such as pembrolizumab, may be a good treatment option for patients with HBsAg+.

HBV may lead to the occurrence of DLBCL via the PD1/PD-L1 cell signaling pathway. Previous studies have shown that HBV infection is closely related to the development of DLBCL [23–25], but most of those data are from retrospective studies or epidemiological investigations rather than from basic experiments. Recently, Zhu J and colleagues found direct evidence of HBV-induced DLBCL in a study that highly suggests that the association of HBV with DLBCL might arise from HBV antigen-selected B cells [9]. In the present study, we found high levels of PD1 expression in DLBCL patients with HBsAg+. However, some studies have reported that the overexpression of PD1 on TILs in the microenvironment may result in T cell exhaustion and immune escape [21,26]. Therefore, we proposed a two-step hypothesis in which HBV results in the occurrence of DLBCL: first, the sustained chronic infection (HBV infection) leads to the overexpression of PD1 on T lymphocytes, leading to the exhaustion of activated T cells and inhibition of the immune system in the infected area; and second, persistent HBV infection generates mutations in infected B lymphocytes, and when the immune system is compromised, mutated B lymphocytes evolve into DLBCL over a period of time.

In conclusion, we explored the PD1 expression levels and the association between PD1 expression and the clinical prognosis of HBsAg+ DLBCL patients. The results showed high levels of PD1 expression in the HBsAg+ group and poor prognoses of PD1-positive patients in the HBsAg+ group. We speculated that the prognosis of PD1-positive patients could be improved by first-line chemotherapy combined with anti-PD1 antibody therapy, such as pembrolizumab. There were at least two limitations to this study. First, the sample size was not large, and second, this study was conducted at a single center, and the conclusions need to be verified by a multicenter study.

## Supporting information

S1 File Participant-level data. (XLSX)
